# Association between High AMH Levels in PCOS Patients and IVF/ICSI Outcomes: a systematic review and meta-analysis

**DOI:** 10.61622/rbgo/2026rbgo25

**Published:** 2026-05-12

**Authors:** Angélica Martins, Renato Ferreira-da-Silva, Ana Margarida Póvoa

**Affiliations:** 1 University of Porto Faculty of Medicine Porto Portugal Faculty of Medicine of the University of Porto, Porto, Portugal.; 2 RISE-Health Porto Portugal RISE-Health, Porto, Portugal.; 3 University of Porto Faculty of Medicine Department of Community Medicine Porto Portugal Department of Community Medicine, Information and Health Decision Sciences, Faculty of Medicine of the University of Porto, Porto, Portugal.; 4 Local Health Unit of São João CRI Reproductive Medicine Porto Portugal CRI Reproductive Medicine, Local Health Unit of São João, Porto, Portugal.; 5 University of Porto Faculty of Medicine Department of Gynecology-Obstetrics and Pediatrics Porto Portugal Department of Gynecology-Obstetrics and Pediatrics, Faculty of Medicine of the University of Porto, Porto, Portugal.

**Keywords:** Anti-Müllerian hormone, Polycystic ovary syndrome, Assisted reproductive techniques, In vitro fertilization, Intracytoplasmic sperm injection, Live birth rate

## Abstract

**Objective:**

To evaluate the association between high Anti-Müllerian Hormone (AMH) levels and key outcomes of In Vitro Fertilization (IVF)/Intracytoplasmic Sperm Injection (ICSI) in women with Polycystic Ovary Syndrome (PCOS).

**Data sources:**

A systematic search was performed in MEDLINE (via PubMed), Web of Science, and Scopus, on September 17, 2024.

**Selection of studies:**

We included observational studies in PCOS patients stratified by AMH levels that reported any of the IVF/ICSI outcomes of interest, namely: live birth rate, clinical pregnancy rate, miscarriage rate, oocytes retrieved, MII oocytes and fertilization rate.

**Data collection:**

Two reviewers independently extracted data and assessed risk of bias using the National Heart, Lung, and Blood Institute Study Quality Assessment Tool. Discrepancies were resolved by consensus. Data were pooled using meta-analysis when appropriate. Sensitivity analyses were conducted using a leave-one-out approach.

**Data synthesis:**

Thirteen studies met the inclusion criteria. Ten were rated as low risk of bias, two as unclear, and one as high risk, indicating overall fair to strong methodological quality. Women with high AMH levels had a lower live birth rate (odds ratio [OR]: 0.85; 95%CI: 0.71–1.02), which became significant after leave-one-out analysis (OR: 0.80; 95%CI: 0.64–0.99). Clinical pregnancy rates were 2% lower in women with elevated AMH levels (OR: 0.98; 95%CI: 0.82–1.17). Miscarriage rates showed a non-significant 25% increase in women with high AMH levels (OR: 1.25; 95%CI: 0.88–1.76). High AMH levels were associated with more retrieved oocytes in the overall meta-analysis (mean difference: 3.52; 95%CI: 1.70–5.33), with consistent findings in sensitivity analyses. There was no significant difference in fertilization rate between the two groups.

**Conclusion:**

While high AMH levels indicate greater ovarian reserve, they negatively influence reproductive outcomes in PCOS patients. These findings highlight the need for individualized ART strategies and further research to clarify underlying mechanisms.

**PROSPERO**: CRD42024596056

## Introduction

The Anti-Müllerian hormone (AMH), a glycoprotein produced by granulosa cells in preantral and small antral follicles, plays a crucial role in follicular growth and development by inhibiting primordial follicle recruitment and regulating granulosa cell differentiation.^(1)^

AMH has been widely used in assisted reproductive technologies (ART) for estimating ovarian reserve, predicting ovarian response to stimulation, identifying patients at risk for ovarian hyperstimulation syndrome (OHSS), and guiding gonadotropin dosing in women undergoing in vitro fertilization (IVF) and intracytoplasmic sperm injection (ICSI).^(2)^ A simplified diagnostic algorithm by Teede et al.^(3)^ suggests the inclusion of AMH levels as an alternative to ultrasound in adults. However, access to AMH assays remains limited in many countries, including within Brazil's public healthcare system, potentially limiting the widespread clinical application of AMH-based diagnostic algorithms.^(4)^

AMH is considered one of the most reliable predictors of the actual number of primordial follicles. However, the number of available oocytes, especially in those with high AMH concentrations, does not always translate into a high number of fertilized oocytes and well-developed embryos.^(5)^ Previous studies revealed that high AMH may inhibit FSH-stimulated aromatase expression, causing androgen excess and impaired preantral follicle growth, which may hinder the development of selectable follicles. AMH levels are positively correlated with LH, testosterone, and DHEA and are linked to polycystic ovary syndrome (PCOS) severity and androgen excess.^(6-8)^ In PCOS, elevated AMH in follicular fluid and granulosa cells suggests intrinsic follicular changes that may inhibit fertilization.^(9)^ However, the impact of excessive AMH on fertilization and embryo development remains unclear due to limited studies.

PCOS is a common endocrine disorder in women of reproductive age, with a prevalence ranging from 5-18%.^(10)^ Women with PCOS often experience amenorrhea or oligomenorrhea, excessive androgen production inducing clinical signs of hyperandrogenism such as hirsutism and abnormal insulin sensitivity.^(11)^

Serum AMH levels are significantly higher in women with PCOS compared to those with normal ovulatory function.^(12)^ However, the predictive value of AMH for ART outcomes remains controversial as elevated AMH levels in these women are believed to correlate with disease severity, which may impact AMH's ability to assess ovarian reserve accurately and, consequently, confounding an association between AMH and ART outcomes.^(13,14)^ Therefore, although AMH level is a useful indicator of fertility potential, it should be interpreted alongside other factors influencing reproductive outcomes, particularly maternal age, which remains the strongest predictor of ART success, as AMH levels naturally decline with advancing age.^(15)^

To date, only a limited number of studies have explored the influence of elevated serum AMH levels on IVF/ICSI outcomes, and their findings have been inconsistent. While some studies have identified a negative effect,^(16-18)^ others have observed a positive impact on pregnancy or live birth outcomes.^(19,20)^

This review aims to synthesize the literature on the relationship between elevated AMH levels and ART outcomes in women with PCOS. By comparing women with high AMH levels to those with low/normal AMH levels, this study seeks to provide a detailed understanding of how varying AMH levels influence key outcomes of medically assisted reproduction, focusing on live birth rate and other relevant IVF/ICSI success indicators.

## Methods

This systematic review and meta-analysis was conducted in accordance with *the Cochrane Handbook for Systematic Reviews of Intervention*^(21)^ and follows the recommendation outlined in the *Preferred Reporting Items for Systematic Reviews and Meta-Analyses (PRISMA)* guidelines.^(22)^ The protocol was registered in PROSPERO (CRD42024596056).

### Eligibility criteria

Studies were eligible for inclusion if they met the following criteria: observational studies, including cohort studies or case-control designs. The study population consisted of women of reproductive age with PCOS undergoing IVF or ICSI. Eligible studies had to measure serum AMH levels before ovarian stimulation and report IVF or ICSI outcomes, such as live birth rate, clinical pregnancy rate, miscarriage rate, number of oocytes retrieved, number of mature (MII) oocytes, or fertilization rate. Additionally, studies were required to stratify their populations based on AMH levels, categorizing participants into low, normal, and high or equivalent thresholds. Exclusion criteria included non-human studies, reviews, commentaries, or editorials, and studies that did not provide primary data or involve quantitative analysis were excluded from this review. Studies were also excluded if they did not assess IVF or ICSI outcomes, did not define high AMH levels using specific cut-off values, or focused on oocyte donation programs. No restrictions were applied regarding the language of publication. The search was limited to studies published between 2014-2024, considering the existence of previous reviews covering earlier periods.

### Search strategy

A comprehensive literature search was conducted across three electronic databases: MEDLINE (via PubMed), Web of Science, and Scopus on September 17, 2024. Our search strategy is available in Supplementary Material – Table1S. In addition to database searches, we examined the reference list of included studies to identify further potential studies.

### Study selection

Two reviewers carried out study selection independently, initially through title/abstract screening (A.M. and A.M.P.) and subsequently through full-text reading (A.M. and A.M.P.). Rayyan QCRI, a systematic review management software (http://rayyan.qcri.org), was used to expedite the screening of abstracts, further eliminate duplicates and facilitate the selection and organization of full-text articles for inclusion.

### Data extraction

Data extraction was conducted independently by two reviewers (A.M. and A.M.P.) using a purpose-built online form. In addition, data were collected on the reported outcomes. The studies were subsequently included in the meta-analysis. Disagreements at any stage of the review process were resolved by consensus.

### Risk assessment

The risk of bias for each included study was independently assessed by two researchers (A.M. and A.M.P.) using the *National Heart, Lung, and Blood Institute Study Quality Assessment Tool* for observational cohort and cross-sectional studies.^(32)^ These tools evaluated key methodological domains, including research question clarity, population definition, sample size justification, exposure and outcome assessment, blinding, follow-up rates, and adjustment for confounders. Each study received an overall quality rating of "good," "fair," or "poor," determined by adherence to these criteria and the resulting risk of bias. Discrepancies between reviewers were resolved by consensus or, when necessary, by consulting a third assessor to ensure accuracy and consistency. The Robvis tool was employed to visually represent the risk of bias assessments and generate summary plots for transparency and clarity in reporting.^(33)^

### Synthesis of data

A narrative synthesis was employed to summarise the data extracted from the included studies, with a focus on highlighting key similarities and differences.

A meta-analysis was conducted separately for each variable of interest. For continuous outcomes, mean differences (MD) and median differences (MedD) with 95% confidence intervals (95% CI) were used. For categorical outcomes, odds ratios (OR) and risk differences (RD) with 95% CI were calculated based on raw data extracted from the primary studies. A continuity correction of 0.5 was applied to OR estimation whenever a cell had a zero count to ensure statistical validity.

Both common-effect and random-effects models were considered based on the level of heterogeneity (I²) and the methodological and study characteristics. Between-study variability was quantified using the I² statistic and the τ² parameter, the latter estimated through the Restricted Maximum Likelihood (REML) method. Heterogeneity was assessed using the Q-Cochran test (p-value) and the I² statistic, with I² > 40% and p-values < 0.10 considered indicative of severe and significant heterogeneity, respectively.

Sources of heterogeneity were assessed using a leave-one-out sensitivity analysis, applied exclusively to study groups with severe/significant heterogeneity (I² > 40%), in which each study was sequentially removed to evaluate its influence on the overall pooled estimate. The overall results of the meta-analysis were visualized using forest plots.

### Statistical analysis

All statistical analyses were conducted in R (version 2023.06.1+524, R Foundation for Statistical Computing, Vienna, Austria) using the *meta* and *metafor* packages.

## Results

**Figure 1:**
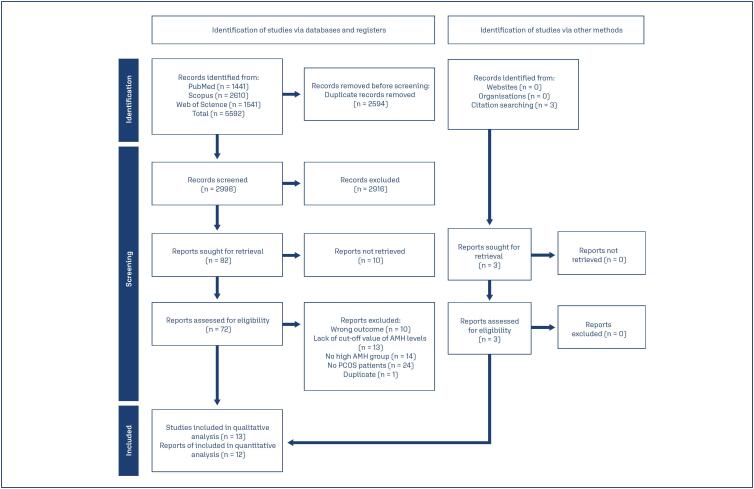
Flow-diagram according to PRISMA statement regarding the process of identification and selection of the studies

After the database search and duplicate removal, a total of 2,998 studies were screened. Following two screening phases a total of 13 published articles were included.^(13,16,17,19,23-31)^. The study selection process is illustrated in figure 1. Reasons for exclusion are presented in Supplementary Material - Table 2S.

A total of 13 primary studies, comprising 12 retrospective cohorts^(13,16,17,19,23-30)^ and one cross-sectional study,^(31)^ were included in the systematic review. (Chart 1)The studies were conducted across diverse geographic regions, such as North America,^(13)^ Europe,^(28)^ Asia^(16,19,26,27,29-31)^ and Africa,^(23,24)^ with sample sizes ranging from 55^(31)^ to 2707^(25)^ participants. The mean age of the participants was 30.49 years. All participants were women with PCOS diagnosed based on the Rotterdam criteria, undergoing IVF or ICSI. Serum AMH levels were assessed using various methods, including enzyme-linked immunosorbent assays (ELISA) and chemiluminescent immunoassays, with considerable variation in AMH cut-off values. For instance, some studies categorized AMH levels based on percentiles,^(16,19,28)^ others in "low", "normal" and "high" categories,^(17,27)^ and others applied one absolute threshold.^(13,23-26,29-31)^ The high AMH levels thresholds or equivalent ranged from 3.0 ng/mL to 14.30 ng/mL (Chart 2).

**Chart 1:** Description of primary studies included in the systematic review, organised by publication year

Study (Author; year)	Journal	Eligibility criteria	Participants number	Age range [mean (SD) or median (IQR)*]	Study left	Inclusion period
**Retrospective cohort studies**						
Tal et al. (2014)^(13)^	American Journal of Obstetrics and Gynecology	Patients with random serum AMH level >5 ng/mL.	n= 134	30.55 (4.67)	Maimonides Medical Left, Brooklyn, NY	April 2009 - May 2013
Kamel et al. (2018)^(23)^	The Journal of Maternal-Fetal & Neonatal Medicine	Patients aged 20–39 years, diagnosed as PCOS according to Rotterdam criteria. Patients with endometriosis, pelvic congestion, large fibroids, untreated endocrine dysfunctions affecting ovulation, or marked biochemical hyperandrogenemia indicative of Cushing's syndrome or congenital adrenal hyperplasia were excluded.	n=976	29.20 (5.34)	Kasr Alainy hospital, Cairo University	April 2013 - June 2016
Tal et al. (2020)^(17)^	Reproductive biology and endocrinology: RB&E	PCOS patients who underwent their first fresh IVF/ICSI cycle aged 18-40 years old. Frozen embryo transfers, patients enrolled in an oocyte donation or gestational surrogacy program or undergoing PDG were excluded.	n= 184	32.04 (4.16)	Not reported	April 2009- April 2014
Guo et al. (2021)^(16)^	Frontiers in Endocrinology	PCOS patients (Rotterdam criteria) undergoing first fresh IVF/ICSI cycles with autologous oocytes. Patients enrolled in an oocyte donation program and undergoing PGD or PGS were excluded.	n= 2436	28.97 (3.40)	Reproductive Medicine Left of Tongji Hospital	January 2016- December 2019
Mohamed et al. (2022)^(24)^	Journal of human reproductive sciences	PCOS patients (Rotterdam consensus criteria) aged 18–40 years who underwent fresh IVF/ICSI cycles. Cases with potential endometrial pathology detected via ultrasound or previously treated surgically, as well as women who underwent frozen cycles or PDG, were excluded from the study.	n= 102	27.25 (4.63)	Women Health Hospital IVF Centre, Assuit University, Egypt.	January 2016- December 2021
Liu et al. (2022)^(19)^	BMC pregnancy and childbirth	Patients ≤ 40 years who presented for their first treatment. Women treated with GnRH antagonist COH protocols, and cycles lacking embryo information or clinical pregnancy data, as well as patients with chromosomal abnormalities, intrauterine death, medical abortion, stillbirth, or ectopic pregnancy, along with patients whose serum AMH measurements were not acquired within 12 months prior to their IVF treatment, were excluded from this study.	Total (n)= 2973 PCOS (n)= 418	31 (29–34) *	Shenzhen Zhongshan Urology Hospital	January 2014- March 2018
Acharya et al. (2022)^(25)^	F&S reports	Women aged <44 years with an AMH level of ≥ 5ng/mL. The exclusion criteria were PDG, planned oocyte/embryo banking, missing AMH level value, and cycles with AMH levels >100 ng/mL.	Total (n)= 10615 PCOS (n)= 2707	31.2 (3.7)	The Society for Assisted Reproductive Technology (SART) Clinical Outcomes Reporting System (CORS)	2012- 2014
Du and Cao (2022)^(26)^	Pakistan Journal of Zoology	Patients diagnosed with PCOS, undergoing IVF-ET at the hospital, and having complete clinical data with cooperation in treatment and follow-up. Patients with congenital immunodeficiency or severe infectious diseases, mental disorders or poor mental status, malignant tumors or severe cardiovascular and cerebrovascular diseases, and severe abnormalities in heart, liver, kidney, or hematopoietic functions were excluded.	n=200	29.47 (2.15)	Reproductive Medicine Left, Second People's Hospital of Jingmen City, China	January 2016 - January 2020
Jabarpour et al. (2023)^(27)^	Clinical laboratory	Patients aged 20 - 45 who underwent their first fresh autologous non-PGD ICSI cycles. Patients with severe male factors, multiple diagnoses, and those with donation oocytes, in vitro-matured oocytes, and oocytes from cancer patients or hepatitis patients were excluded.	Total (n)= 881 PCOS (n)= 501	34 (30, 37) *	Infertility clinic of Dr. Shariati Hospital affiliated with Theran University of Medical Sciences (TUMS), Theran, Iran.	2012- 2020
Vale-Fernandes et al. (2023)^(28)^	Biomedicines	Women had to have a PCOS (Rotterdam criteria) and who underwent IVF/ICSI cycles with autologous oocytes	n=150	33.04 (3.75)	Single academic public left, Porto, Portugal	January 2018- December 2022
Su et al. (2023)^(29)^	Frontiers In Endocrinology	Patients with PCOS (Rotterdam criteria) aged 18-39 years undergoing ET cycles. Patients with repetitive initiated cycles, treatment without the GnRH antagonist protocol, history of ovarian surgery, congenital or acquired reproductive malformations, recurrent miscarriages, endometriosis, and uncontrolled hypertension, diabetes, and thyroid diseases were excluded.	n= 344	29 (26, 32) *	Reproductive Health and Infertility Department, Guangdong Women and Children Hospital, China	November 2014 - September 2018
Huang et al. (2023)^(30)^	Reproductive biology and endocrinology: RB&E	Patients with age 20–39 years, BMI <30 kg/m², first IVF/ICSI treatment with the long GnRH agonist protocol, controlled ovarian stimulation with recombinant FSH, and a PCOS diagnosis based on the Rotterdam criteria. Patients diagnosed with any type of endometriosis, or with a history of ovary surgery, any untreated endocrine dysfunction e.g., hypothyroidism/ hyperthyroidism, or hyperprolactinemia, and marked biochemical hyperandrogenemia were excluded	n= 825	30.2 (3.8)	Department of Reproductive Medicine at Women and Children's Hospital, School of Medicine, Xiamen University.	January 2019 - December 2021
**Cross-sectional study**						
Jaralla et al. (2024)^(31)^	Journal of Obstetrics, Gynecology and Cancer Research	Patients with PCOS undergoing in vitro fertilization cycles	n=55	NR	Baghdad and Al-Najaf infertility lefts	June 2022 - June 2023

**Chart 2:** Ovarian stimulation protocols, AMH assessment (methods and cut-offs) in included studies

Study (Author; year)	Ovarian stimulation protocol	AMH assay method	AMH cut-off (ng/mL)
Tal et al. (2014)^(13)^	GnRH agonist in a long protocol or a GnRH antagonist.	ELISA	5-10 ng/mL >10-14 ng/mL
Kamel et al. (2018)^(23)^	GnRH antagonist	Gen II ELISA kit	Group A (AMH ≤4.6 ng/mL) Group B (AMH >4.6 ng/mL)
Tal et al. (2020)^(17)^	GnRH agonist or a GnRH antagonist	ELISA	Low (< 3.32 ng/mL) Average (3.32–8.27 ng/mL) High (8.27 ng/mL)
Guo et al. (2021)^(16)^	GnRH agonist or antagonist treatment	ELISA, AMH ELISA kit	<25th percentile (≤ 6.77 ng/mL) 25-75th percentile (6.77-14.30 ng/mL) >75th percentile (≥14.30 ng/mL)
Mohamed et al. (2022)^(24)^	GnRH antagonist protocol	VIDAS automated AMH assay- ELISA	normal/moderately elevated (<7.0 ng/mL) markedly elevated AMH (≥7.0 ng/mL)
Liu et al. (2022)^(19)^	GnRH agonist protocol	CLIA under Cobas e601	0-25th percentage (≤ 4.91 ng/mL) 25th-75th percentage (4.91- 10.88 ng/mL) 75th-100th percentage (> 10.88 ng/mL)
Acharya et al. (2022)^(25)^	NR	NR	AMH ≤ 12 ng/mL AMH > 12 ng/mL
Du and Cao (2022)^(26)^	GnRH agonist (triptorelin)	ELISA	Group A (< 6.99 ng/mL) Group B (≥ 6.99 ng/mL)
Jabarpour et al. (2023)^(27)^	GnRH agonist (n=101) or antagonist (n=780)	ELISA	low (≤ 0.3 - 0.9 ng/mL) normal (≥ 1.0 ng/mL) high (≥ 3.0 ng/mL).
Vale-Fernandes et al. (2023)^(28)^	GnRH antagonist protocol	Not reported	<25th percentile (< 3.7 ng/mL) 25th-75th percentile (3.7–7.4 ng/mL) >75th percentile (> 7.4 ng/mL)
Su et al. (2023)^(29)^	GnRH antagonist protocol	NR	1 ng/mL ≤ AMH < 12 ng/mL 12 ng/mL ≤ AMH < 25 ng/mL
Huang et al. (2023)^(30)^	Long-acting GnRH agonist (triptorelin)	CLIA kit	AMH ≤9.30ng/mL AMH >9.3ng/mL
Jaralla et al. (2024^)(31^)	NR	NR	Group1 (2.25-5.71 ng/mL) Group 2 (> 5.71 ng/mL)

ELISA: Enzyme-linked immunosorbent assay; CLIA: Chemiluminescent Immunoassay; NR: Not Reported

**Figure 2:**
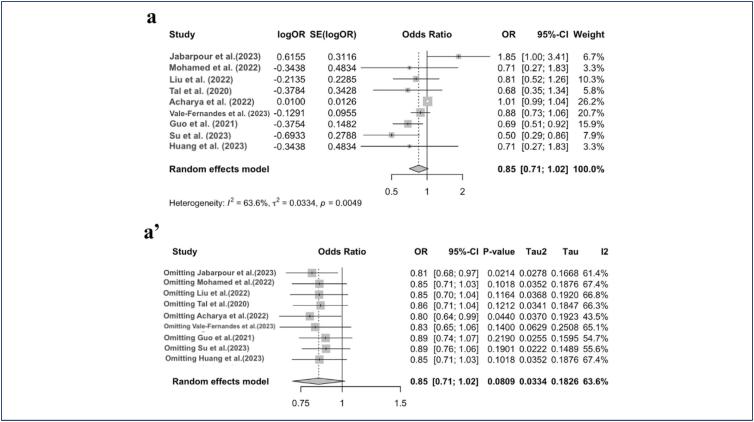
Live Birth Rate

Nine studies were initially included in the meta-analysis for live birth rate.^(16,17,19,24,25,27-30)^ The pooled OR for live birth was 0.85 (95%CI: 0.71–1.02; p=0.0809), suggesting a 15% reduction in the probability of live birth in women with elevated AMH levels compared to those with normal or low levels, though this difference was not statistically significant (Figure 2). However, high heterogeneity was observed (I²=63.6%; p=0.0049). A leave-one-out sensitivity analysis identified Acharya et al.^(25)^ as the main contributor. Upon its exclusion, the heterogeneity decreased to a moderate level (I²=43.5%; p=0.089), and the pooled OR shifted to 0.80 (95%CI: 0.64–0.99; p=0.044), indicating a statistically significant 20% reduction in the live birth rate among women with high AMH levels compared to those with normal or low levels.

Forest plot of pooled OR, 95% CI, Random-Effects Model. (a′) Leave-one-out sensitivity analysis

**Figure 3:**
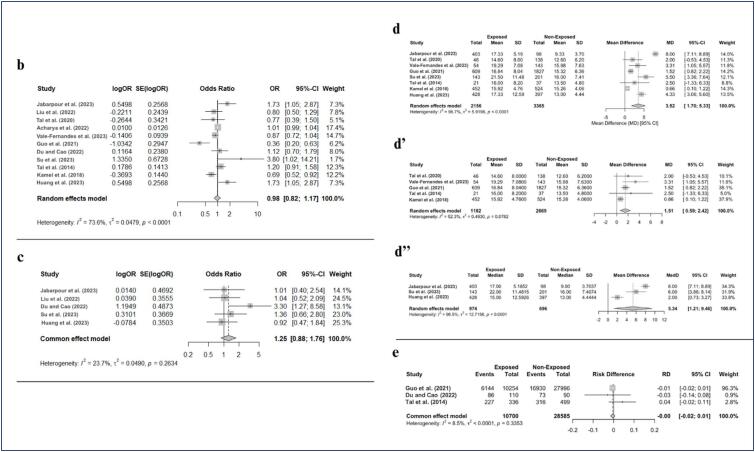
(b) Clinical Pregnancy- Forest plot of pooled OR, 95% CI, Random-Effects Model. (c) Miscarriage Rate- Forest plot of pooled OR, 95% CI, Common-Effects Model. (d) Number of Oocytes Retrieved: (d) All studies: forest plot of pooled MD, 95% CI, Random-Effects Model; (d’) Studies reporting means: forest plot of pooled MD, 95% CI, Random-Effects Model; (d") Studies reporting medians: forest plot of pooled MedD, 95% CI, Random-Effects Model. (e) Fertilization Rate- Forest plot of pooled RD, 95% CI, Common-Effects Model

Clinical pregnancy rates were analyzed in 11 studies.^(16,17,19,23-30)^ The pooled OR was 0.98 (95%CI:0.82–1.17; p=0.7896), indicating a reduction of 2% in the clinical pregnancy rate in women with elevated AMH levels compared to those with low or normal levels (Figure 3). Heterogeneity was high (I²=73.6%; p < 0.0001), but leave-one-out sensitivity analysis showed that no single study disproportionately impacted the overall estimate.

Jaralla et al.^(31)^ was excluded due to their cross-sectional design. However, among women with high AMH, 40% achieved clinical pregnancy versus 25% with normal AMH. Despite limitations, these findings suggest a positive association between high AMH and clinical pregnancy, contrary to the meta-analysis.

Five studies reported miscarriage rates.^(19,26,27,29,30)^ The pooled OR was 1.25 (95%CI:0.88–1.76; p=0.2131), suggesting a non-significant 25% increase in miscarriage among women with high AMH levels. Heterogeneity was low (I²=23.7%; p=0.2634). One study^(26)^ reported a markedly higher miscarriage risk (OR: 3.30; 95%CI: 1.27–8.58; p=0.2482).

Eight studies analyzed the number of oocytes retrieved.^(13,16,17,23,27-30)^ Five studies reported the outcome as a mean with standard deviation,^(13,16,17,23,28)^ and three studies presented it as a median with interquartile range.^(27,29,30)^ To standardize the analysis, the medians and interquartile ranges were converted into means and standard deviations using the method proposed by Hozo et al.^(34)^ The random-effects meta-analysis, incorporating all studies, yielded a pooled MD of 3.52 (95%CI: 1.70–5.33; p < 0.0001), indicating that women with high AMH levels retrieved, on average, 3.52 more oocytes than those with normal or low AMH levels. However, substantial heterogeneity was observed (I²=96.7%; p < 0.0001), warranting further sensitivity analyses. A leave-one-out sensitivity analysis demonstrated the robustness of the findings, as the overall MD remained within the same range (2.72–4.01) regardless of which study was excluded. The heterogeneity remained high across all iterations, suggesting a cumulative effect of multiple studies.

Given the potential impact of median-to-mean conversions on the results, a subgroup analysis was conducted excluding the three studies that initially reported medians. This yielded a pooled MD of 1.51 (95%CI: 0.59–2.42), with a reduction in heterogeneity (I²=52.3%; p=0.0782). A separate meta-analysis, including only the three studies that initially reported medians, was also performed, showing a pooled MedD of 5.34 (95%CI: 1.21–9.46), with substantial heterogeneity (I²= 96.5%; p < 0.0001). This suggests that the inclusion of converted medians may have led to overestimating the effect size.

Overall, the findings indicate that women with high AMH levels retrieve more oocytes than those with lower levels.

Fertilization rate was reported in six studies,^(13,16,17,26-28)^ but only three^(13,16,26)^ were included in the meta-analysis due to the absence of necessary data in the remaining studies. The pooled RD was -0.00 [95%CI: -0.02; p=0.01], with low heterogeneity (I²=8.5%; p=0.3353), indicating no significant difference in fertilization rate between the two groups. Additionally, three studies^(17,27,28)^ reported fertilization rates between 63.34% to 80% in the high AMH group and 58% to 89% in the normal/low AMH group. These findings are consistent with the meta-analytic results, but the lack of dispersion measures prevented their inclusion in the quantitative analysis.

Four studies analyzed the number of MII oocytes.^(13,16,24,27)^ Jabarpour et al.^(27)^ reported this outcome as a median in the high AMH group - 13(10, 17) - and in the low/normal group - 7(5,9). Guo et al.^(16)^ reported a mean number of 14.83±7.58 in the high AMH group and 13.41±5.81 in the normal/ low AMH group. Mohamed et al.^(24)^ reported a significant positive correlation between AMH concentration and the number of MII oocytes (Spearman r=0.276; p=0.006). Finally, Tal et al.^(13)^ presented the absolute number of 336 MII oocytes across the 21 cycles performed in the high AMH group and 499 MII oocytes across the 37 cycles of the normal AMH group.

**Figure 4:**
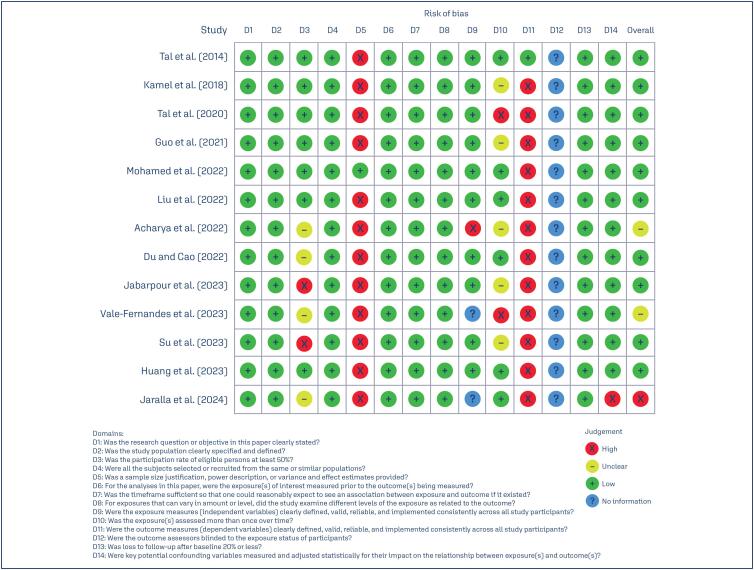
Global traffic light for risk of bias of all observational studies included in the systematic review, displayed by article. "Low" risk of bias = ≥75% "Yes" responses, with no critical methodological flaws (e.g., exposure measured before outcome, valid measures, appropriate control for confounders). "Unclear" risk of bias = 50–74% "Yes" responses, with some methodological limitations, including up to one critical flaw, but still providing useful findings. "High" risk of bias = <50% "Yes" responses or ≥2 critical flaws (e.g., exposure assessed after outcome, unreliable measures, no adjustment for confounders), indicating high risk of bias and reduced validity

**Figure 5:**
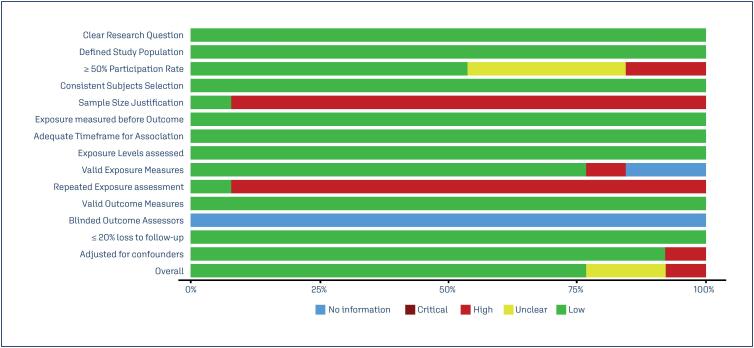
Global summary plot for risk of bias of all included observational studies, displayed by item

The selected articles’ bias risk is displayed in figures 4 and 5. Among the included studies, ten were classified as having a low risk of bias,^(13,16,17,19,23,24,26,27,29,30)^ indicating strong methodological quality. Two studies^(25,28)^ had an unclear risk of bias due to methodological limitations, while one^(31)^ was rated as having a high risk of bias, suggesting significant concerns regarding its validity. The items most frequently associated with a high risk of bias included consistent subject selection, valid exposure measures, and repeated exposure assessment. These domains exhibited a notable proportion of studies classified as unclear or high risk, indicating methodological weaknesses.

## Discussion

The findings of this systematic review and meta-analysis contribute to the ongoing debate on the impact of AMH levels on IVF and ICSI outcomes in women with PCOS. Our results confirm that elevated serum AMH level is inversely associated with live birth and clinical pregnancy rates while also being linked to higher miscarriage rates in this population, supporting previous research on the detrimental effects of excessive AMH on reproductive success.^(5,35)^ Additionally, we found that high AMH levels correlate with an increased number of oocytes retrieved and similar fertilization rates; similar results were reported by Yuwen et al.^(35)^ These findings emphasize the potential role of AMH as a prognostic biomarker in ART for PCOS patients and highlight the need for personalized treatment strategies.

Our meta-analysis demonstrated significantly higher LBR in women with low/normal AMH than those with high AMH. A similar trend was observed for CPR, though not statistically significant. These results diverge from studies in the general population, where higher AMH levels are often correlated with better ART outcomes.^(20,36,37)^ Previous meta-analyses indicated AMH´s moderate predictive value for clinical pregnancy in a general IVF population, whereas in PCOS patients, the predictive utility appears to be different.^(38)^ The weaker predictive ability of AMH for pregnancy outcomes in women with PCOS may result from its close association with disease severity, as elevated levels correlate with the three PCOS hallmarks—polycystic ovarian morphology, oligo/anovulation, and hyperandrogenism— thereby confounding its role as a predictor of ART success.^(39,40)^

Vale-Fernandes et al.^(28)^ and other studies observed an inverse relationship between FSH and AMH levels and a positive trend between LH and AMH in PCOS women.^(16,18,28)^ These findings support the role of elevated AMH in disrupting folliculogenesis through two key mechanisms. First, it inhibits FSH-dependent dominant follicle selection, reducing granulosa cell sensitivity to FSH and potentially causing follicular arrest. Second, AMH is positively correlated with hyperandrogenemia, suppressing FSH-stimulated aromatase mRNA expression in granulosa-luteal cells, thereby reducing aromatase activity and inducing intraovarian androgen accumulation.^(17,28,41)^ Androgens, in turn, promote early follicular growth independently of FSH, further increasing AMH production. In fact, AMH levels in PCOS women are 2–4 times higher than in those without the condition, primarily due to granulosa cells within individual follicles overproducing AMH by up to 75 times the normal amount rather than a higher follicle count.^(42-44)^ Tal et al.^(17)^ also found that total testosterone levels were significantly higher in the high serum AMH group compared to the average/low AMH groups, highlighting the complex relationship between AMH levels and ART outcomes across different PCOS phenotypes, with hyperandrogenic phenotypes in PCOS women shown to be associated with poorer CPR and LBR.^(17)^

Our results also found a non-significant 25% increase in miscarriage rates among women with high AMH levels. Beyond oocyte-related factors, the detrimental effects of high AMH and androgens on endometrial homeostasis and receptivity may further explain implantation failure and recurrent miscarriage in women with PCOS.^(16,28)^

In our study, women with high AMH levels showed a trend toward an increased number of oocytes retrieved, consistent with previous research findings. However, this increase did not translate into improved clinical pregnancy rate although there were no differences in fertilization rates. One possible explanation is that while elevated AMH levels indicate a greater ovarian reserve, they are often associated with a decline in oocyte quality.^(38)^ As a result, the higher number of oocytes retrieved leads to a larger pool of embryos for selection but does not necessarily enhance clinical pregnancy rate. Several studies have suggested that higher AMH levels are associated with lower oocyte maturity rates.^(11,45)^ Dai et al.^(46)^ reported that older women with lower AMH levels had a higher proportion of MII oocytes, possibly due to differences in follicular development. Compared with a large cohort of developing follicles, a smaller number of oocytes may receive adequate nutritional support from the ovary, promoting their maturation.^(46)^ Despite this, our findings do not show a decrease in MII oocytes among women with high AMH levels. On the contrary, our results reported a positive association between higher AMH levels and the number of mature oocytes.^(16,27)^ This discrepancy may point to differing sample characteristics or methodological factors that could influence outcomes related to oocyte maturity.

This study is the most recent and comprehensive systematic review and meta-analysis assessing the impact of high AMH levels on ART outcomes in women with PCOS. The inclusion of studies from diverse geographic regions enhances the generalizability of findings. Additionally, rigorous methodological approaches in compliance with recent guidelines,^(22)^ including risk-of-bias assessment, sensitivity analyses, and stratified meta-analyses, strengthen the reliability of the results. Although the process of systematic literature review and meta-analysis provide stronger effect estimates with reduced random error, it does come with limitations. Overall, while most included studies demonstrated low risk of bias and strong methodological quality, the presence of studies with unclear or high risk highlights potential limitations in the consistency of subject selection and exposure assessment, which should be considered when interpreting the results. First, the included studies reported different effect measures, and our pooled analysis for each outcome did not incorporate data from all eligible studies, which may have introduced bias. Second, some studies did not report adjusted effect estimates, which are less prone to confounding than crude estimates - the measures used in our meta-analysis. Third, the variation in study populations, including differences in baseline characteristics such as age, BMI, and clinical conditions, and differences in stimulation protocols and AMH assays, including enzyme-linked immunosorbent assays and chemiluminescent immunoassays, with considerable variation in AMH cut-off values, may have contributed to the observed heterogeneity; however, a universal threshold for AMH is not clinically appropriate, which further underscores the variability across studies. As in the study by Acharya et al.,^(25)^ the observed heterogeneity may also be explained by several methodological aspects, including its retrospective cohort design based on the large multicenter SART-CORS database, in contrast to the predominantly unicentric cohorts of the other studies. Additional contributing factors include its unclear risk of bias, the use of exposure measures not consistently or clearly defined across participants, statistical differences in analysis, and its comparatively larger sample size, all of which may have amplified variability relative to the remaining studies. Lastly, the limited number of studies and small sample sizes may have reduced the statistical power, further constraining the interpretation of the findings.

## Conclusion

Our study confirms that high AMH levels are associated with lower live birth and clinical pregnancy rates in women with PCOS, alongside a higher risk of miscarriage. While elevated AMH correlates with a greater number of retrieved oocytes, it does not improve pregnancy rate, raising concerns about oocyte quality. These findings underscore the need for individualized ART strategies, including optimized stimulation protocols (milder), ‘freeze-all’ and tailored patient counseling, to improve outcomes in PCOS patients with high AMH. Further research is required to refine AMH-based prognostic models and understand its impact on both oocyte and endometrial quality, with particular attention to tailoring stimulation protocols according to PCOS phenotypes. Dedicated studies specifically designed to address these aspects will be crucial to advancing personalized approaches and improving reproductive outcomes in women with PCOS.

## Data Availability

The research data are described in the article presented.
