# Supplementary material for: Association between High AMH Levels in PCOS Patients and IVF/ICSI Outcomes: a systematic review and meta-analysis
Source: Rev Bras Ginecol Obstet. 2026 May 12;48:e-rbgo25. doi: 10.61622/rbgo/2026rbgo25 (PMC13399490; doi:10.61622/rbgo/2026rbgo25)
Supplement: Supplementary Material [file 1806-9339-rbgo-48-e-rbgo25-Supp01.pdf]

Supplementary material

Table S 1. Queries used for searching MEDLINE/PubMed, Web of Science and Scopus.

DATABASE	SEARCH
MEDLINE (PubMed)	(“AMH”[Text Word] OR “Anti-Müllerian Hormone”[MeSH] OR “Müllerian inhibiting factor”[Text Word] OR “Ovarian reserve”[Text Word])<br><br>AND<br><br>(“Fertilization in vitro”[MeSH] OR “IVF”[Text Word] OR “ICSI”[Text Word] OR “ Sperm Injections, Intracytoplasmic “[MeSH] OR “Reproductive Techniques, Assisted”[MeSH] OR “ART”[Text Word] OR “Assisted reproduction”[Text Word])<br><br>AND<br><br>(“Oocyte retrieval”[Text Word] OR “Embryo quality”[Text Word] OR “Pregnancy rate”[Text Word] OR “Clinical pregnancy”[Text Word] OR “Live birth”[MeSH] OR “Implantation rate”[Text Word])
Web of Science	(“AMH” OR “Anti-Müllerian Hormone” OR “Müllerian inhibiting factor” OR “Ovarian reserve”)<br>AND<br>(“In Vitro Fertilization” OR “IVF” OR “ICSI” OR “Intracytoplasmic Sperm Injection” OR “Assisted Reproductive Technology” OR “ART” OR “Assisted reproduction”)<br>AND<br>(“Oocyte retrieval” OR “Embryo quality” OR “Pregnancy rate” OR “Clinical pregnancy” OR “Live birth” OR “Implantation rate”) (All Fields)
Scopus	TITLE-ABS-KEY (“AMH” OR “Anti-Müllerian Hormone” OR “Müllerian inhibiting factor” OR “Ovarian reserve”)<br>AND<br>TITLE-ABS-KEY (“In Vitro Fertilization” OR “IVF” OR “ICSI” OR “Intracytoplasmic Sperm Injection” OR “Assisted Reproductive Technology” OR “ART” OR “Assisted reproduction”)<br>AND<br>TITLE-ABS-KEY (“Oocyte retrieval” OR “Embryo quality” OR “Pregnancy rate” OR “Clinical pregnancy” OR “Live birth” OR “Implantation rate”)
TIME LIMIT	2014 - 2024

Table S 2. List of references excluded during the full-text review phase, categorized by reason for exclusion

First author	Journal; Year of publication	Title
		**Wrong outcome**
Goswami et al.	Journal of human reproductive sciences; 2017	Is AMH Level, Independent of Age, a Predictor of Live Birth in IVF?
Hamdine et al.	Fertility and sterility; 2015	Antimullerian hormone: prediction of cumulative live birth in gonadotropin-releasing hormone antagonist treatment for in vitro fertilization.
Shen et al.	European journal of medical research; 2024	Effects of total gonadotropin dose on embryo quality and clinical outcomes with AMH stratification in IVF cycles: a retrospective analysis of 12,588 patients.
Višnová et al.	Reproductive biomedicine online; 2021	Clinical outcomes of potential high responders after individualized FSH dosing based on anti-Mullerian hormone and body weight.
Kim et al.	Fertility and sterility; 2023	Elevated antimullerian hormone levels are not associated with preterm delivery after inτ ⁺vitro fertilization or ovulation induction.
Melado et al.	Reproductive biomedicine online; 2020	Anti-Mullerian hormone is an independent marker for oocyte survival after vitrification
Messelt et al.	Fertility and sterility; 2021	Live birth differences in women with significantly above average AMH level undergoing IVF
Ulker et al.	Fertility and Sterility; 2024	High antimullerian hormone level in oocyte donors: Is it a problem?
He et al.	Frontiers in Endocrinology; 2023	Serum anti-Mullerian hormone levels are associated with perinatal outcomes in women undergoing IVF/ICSI: A multicenter retrospective cohort study
Aghssa et al.	Reproductive Health; 2015	Optimal cutoff value of basal anti-mullerian hormone in iranian infertile women for prediction of ovarian hyper-stimulation syndrome and poor response to stimulation Female Fertility
		**Lack of cut-off value of AMH levels**
Zebitay et al.	Journal of obstetrics and gynaecology: the journal of the Institute of Obstetrics and Gynaecology; 2017	The role of ovarian reserve markers in prediction of clinical pregnancy.
Morales et al.	JBRA assisted reproduction; 2022	Evaluation of the Anti-Mullerian Hormone and its Association with Embryo Quality in Advanced Reproductive Treatments in a Latin American Population.
Wang et al.	Journal of clinical medicine; 2023	Association of the Cumulative Live Birth Rate with the Factors in Assisted Reproductive Technology: A Retrospective Study of 16,583 Women.
Brodin et al.	Acta obstetricia et gynecologica Scandinavica; 2015	Comparing four ovarian reserve markers-associations with ovarian response and live births after assisted reproduction.
Zhang et al.	Archives of gynecology and obstetrics; 2021	Predictive value of anti-Mullerian hormone on pregnancy outcomes in in-vitro fertilization/ intracytoplasmic single sperm injection patients at different ages.
Scheffer et al.	JBRA assisted reproduction; 2018	Are age and anti-Mullerian hormone good predictors of ovarian reserve and response in women undergoing IVF?
Ye et al.	Drug discoveries & therapeutics; 2023	Higher serum AMH level is associated with better pregnancy outcomes of IVF/ICSI assisted pregnancy in infertile patients under 35 years old.
Borges et al.	JBRA assisted reproduction; 2017	The predictive value of serum concentrations of anti-Mullerian hormone for oocyte quality, fertilization, and implantation.

Continue...

Continuation.

Li et al.	Reproductive biomedicine online; 2016	Anti-Mullerian hormone for prediction of ovarian response in Chinese infertile women undergoing IVF/ICSI cycles: a prospective, multi-centre, observational study.
Ramezanali et al.	Reproductive biomedicine online; 2016	Assisted reproductive outcomes in women with different polycystic ovary syndrome phenotypes: the predictive value of anti-Mullerian hormone.
Ashrafi et al.	Journal of obstetrics and gynaecology : the journal of the Institute of Obstetrics and Gynaecology; 2017	Predictive values of anti-mullerian hormone, antral follicle count and ovarian response prediction index (ORPI) for assisted reproductive technology outcomes.
Wang et al.	Journal of obstetrics and gynaecology; 2015	A higher anti-Mullerian hormone level is associated with an increased chance of pregnancy in patients undergoing controlled ovarian stimulation and intrauterine insemination
Seckin et al.	Journal of the Chinese Medical Association; 2019	The role of anti-Mullerian hormone in prediction of pregnancy in young and older women with unexplained infertility undergoing intrauterine insemination
**No high AMH group**		
Liu et al.	Reproductive health; 2022	Predictive value of anti-Mullerian hormone for pregnancy outcomes following assisted reproductive techniques (ART) in Southwest China.
Dai et al.	Scientific reports; 2020	AMH has no role in predicting oocyte quality in women with advanced age undergoing IVF/ICSI cycles.
Ezoe et al.	BMC pregnancy and childbirth; 2020	Anti-Mullerian hormone is correlated with cumulative live birth in minimal ovarian stimulation with clomiphene citrate: a retrospective cohort study.
Zheng et al.	Medicine; 2017	Ovarian response prediction in controlled ovarian stimulation for IVF using anti-Mullerian hormone in Chinese women: A retrospective cohort study.
Park et al.	Clinical and experimental reproductive medicine; 2015	Anti-Mullerian hormone levels as a predictor of clinical pregnancy in in vitro fertilization/intracytoplasmic sperm injection-embryo transfer cycles in patients over 40 years of age.
Aydın et al.	Iranian journal of reproductive medicine; 2015	Assessment of the relationship of basal serum anti-mullerian hormone levels with oocyte quality and pregnancy outcomes in patients undergoing ICSI.
Vijay et al.	Journal of reproduction & infertility; 2022	Association Between AMH Levels and Fertility/Reproductive Outcomes Among Women Undergoing IVF: A Retrospective Study.
Liss et al.	Gynecological endocrinology: the official journal of the International Society of Gynecological Endocrinology; 2017	Clinical utility of different anti-Mullerian hormone - AMH assays for the purpose of pregnancy prediction.
Seyedoshohadaei et al.	Medical Science; 2022	The relationship between Anti-Mullerian Hormone and ovarian response and fertility outcome in infertile women undergoing assisted reproductive techniques (ART)
Hyun-kyung Park et al.	International Journal of Gynecology and Obstetrics; 2024	Effect of anti-Mullerian hormone on early pregnancy loss in hormone replacement therapy,Äìfrozen,Äìthawed embryo transfer cycles: An analysis including 6597 pregnant patients undergoing their first inτ ᐩvitro fertilization/intracytoplasmic sperm injection cycle
Heidary et al.	Journal of Reproduction and Infertility; 2024	The Association of AMH Level with the Number and Quality of Oocytes in Women Undergoing IVF/ICSI: A Single-Center Study
Alanazi et al.	Middle East Fertility Society Journal; 2018	Antimullerian hormone (AMH) level and IVF/ICSI cycle outcome in expected poor responders
Jamil et al.	Pakistan Journal of Medical Sciences; 2016	Anti Mullerian hormone: Ovarian response indicator in young patients receiving long GnRH agonist protocol for ovarian stimulation
Lukaszuk et al.	Reproductive Biology; 2014	Anti-Mullerian hormone (AMH) is a strong predictor of live birth in women undergoing assisted reproductive technology
**No PCOS patients**		
Liu et al.	BMC pregnancy and childbirth; 2022	Serum anti-Mullerian hormone levels are associated with early miscarriage in the IVF/ICSI fresh cycle.
Azizi et al.	International journal of gynaecology and obstetrics; 2019	Serum anti-Mullerian hormone is associated with oocyte dysmorphisms and ICSI outcomes.
Gomez et al.	Archives of gynecology and obstetrics; 2016	The influence of AMH on IVF success.
Hou et al.	Scientific reports; 2023	Serum levels of anti-Mullerian hormone influence pregnancy outcomes associated with gonadotropin-releasing hormone antagonist treatment: a retrospective cohort study.
Sun et al.	JBRA assisted reproduction; 2021	High anti-Mullerian hormone levels might not reflect the likelihood of clinical pregnancy rate in IVF/ICSI treatment.
Zhang et al.	Reproductive biology and endocrinology : RB&E; 2019	IVF outcomes of women with discrepancies between age and serum anti-Mullerian hormone levels.
Kien et al.	European journal of obstetrics, gynecology, and reproductive biology; 2022	Anti-Mullerian hormone is a predictor of medium-term cumulative live birth following in vitro fertilization/intracytoplasmic sperm injection: A retrospective study.
Hu et al.	Reproductive biomedicine online; 2020	Association of serum anti-Mullerian hormone and other factors with cumulative live birth rate following IVF.
Pacheco et al.	Reproductive biomedicine online; 2018	Very low anti-mullerian hormone concentrations are not an independent predictor of embryo quality and pregnancy rate.
Amsiejiene et al.	Gynecological endocrinology: the official journal of the International Society of Gynecological Endocrinology; 2017	The influence of age, body mass index, waist-to-hip ratio and anti-Mullerian hormone level on clinical pregnancy rates in ART.
Alson et al.	European journal of obstetrics, gynecology, and reproductive biology; 2018	Anti-mullerian hormone levels are associated with live birth rates in ART, but the predictive ability of anti-mullerian hormone is modest.
Preaubert et al.	Reproductive biomedicine online; 2019	Live birth rates remain stable in modified natural IVF despite low anti-Mullerian hormone: analysis of 638 cycles.
Tsai et al.	Reproductive biomedicine online; 2022	High serum anti-Mullerian hormone concentrations have a negative impact on fertilization and embryo development rates.
Wang al.	Reproductive biomedicine online; 2019	Anti-Mullerian hormone in association with euploid embryo transfer outcomes.
Karacin et al.	Journal of human reproductive sciences; 2023	Is There a Relationship between Serum Anti-Mullerian Hormone Levels and Abortion Rates in Patients Who Received In vitro Fertilisation-Embryo Transfer Cycles?
Umarsingh et al.	PEERJ; 2020	The relationship between anti-Mullerian hormone (AMH) levels and pregnancy outcomes in patients undergoing assisted reproductive techniques (ART)

Continue...

Continuation.

Hafezi et al.	International Journal Of Fertility & Sterility; 2024	Association of Anti-Mullerian Hormone on Oocyte Maturation, Fertilization, and Pregnancy Rates in Patients under Assisted Reproductive Technology Cycles: A Cross-Sectional Study
Li et al.	Current Medical Science; 2018	Basal Serum Anti-Mullerian Hormone Level as a Predictor of Clinical Outcomes in Freezing-all Embryo Transfer Program
Albu et al.	Revista De Chimie; 2019	The Non-linear Association Between Serum Level of Anti-Mullerian Hormone and <i>in vitro</i> Fertilisation Outcome
Mohammed et al.	Iraqi Journal of Science; 2024	Relationship Between Anti-Mullerian Hormone (AMH) and Other Influencing Factors with in Vitro Fertilization (IVF) Outcome of Iraqi Infertile Women
Erdoƒüan et al.	Duzce Medical Journal; 2022	Evaluation of Anti-Mullerian Hormone in Predicting In Vitro Fertilization Cycle Outcomes
Chen et al.	Taiwanese Journal of Obstetrics and Gynecology; 2014	Effects of anti-Mullerian hormone and follicle stimulating hormone levels on invitro fertilization pregnancy rate
Reichman et al.	Fertility and Sterility; 2014	Value of antimullerian hormone as a prognostic indicator of in vitro fertilization outcome
Lehmann et al.	Journal of Assisted Reproduction and Genetics; 2014	Anti-Mullerian hormone (AMH): A reliable biomarker of oocyte quality in IVF
	**Duplicate**	
Tal et al.	Reproductive Sciences; 2015	High Serum Antimullerian Hormone (AMH) Concentrations Are Associated With Lower Live Birth Rates in Polycystic Ovarian Syndrome (PCOS) Women Undergoing Assisted Reproductive Technology (ART).

Table S 3. Summary of Meta-Analytic Results

Outcome	No. of studies	Pooled effect size; 95% CI; p-value	I^2 (%)
Live Birth			
All studies	9	OR=0.85; 95% CI: 0.71–1.02; p= 0.0809	63.6%
Omitting Acharya 2022	8	OR=0.80; 95% CI: 0.64–0.99; p= 0.044	43.5%)
Clinical pregnancy	11	OR = 0.98; 95% CI: 0.82-1.17; p = 0.79	73.6%
Miscarriage	5	OR= 1.25; 95%CI:0.88–1.76; p=0.2131	23.7%
Oocytes retrieved			
All studies	9	MD = 3.52; 95% CI: 1.70-5.33; p < 0.001	96.7%
Only means	6	MD = 1.51; 95%CI:0.59; 2.42; p = 0.001	52.3%
Only medians	3	MedD = 5.34; 95%CI:1.21–9.46; p = 0,01	96.5%
Fertilization	3	RD = -0.00; 95% CI: -0.02-0.01; p = 0.59	8.5%

MD: mean difference; RD: risk difference; OR: odds ratio; MedD: median difference; CI: confidence interval.
